# Stones in genomic storms: transposable element-driven variation in *Prunus* fruit trees

**DOI:** 10.1186/s13100-026-00403-1

**Published:** 2026-05-12

**Authors:** Attila Hegedűs, Beti Ivanovska, Júlia Halász

**Affiliations:** Institute of Genetics and Biotechnology, Department of Plant Biotechnology, Horticultural Plant Genetics Group, Hungarian University of Agriculture and Life Sciences, Ménesi út 44, Budapest, 1118 Hungary

**Keywords:** Genome evolution, *Prunus*, Regulation, Molecular markers, Mutations, Phenotypic diversity, Retrotransposons, Self-incompatibility, Transposable elements, Transposons

## Abstract

Transposable elements (TEs) are major components of plant genomes and drivers of structural and functional diversification. In *Prunus* species, including peach, almond, apricot, cherry, and plum, TEs constitute more than half of the genome and shape evolutionary trajectories and phenotypic traits. This review synthesizes knowledge on TE composition, evolutionary dynamics and functional impacts in *Prunus*, highlighting advances enabled by long-read sequencing and structure-aware annotation pipelines. Both class I retrotransposons and class II DNA transposons contribute to genome expansion, gene duplication, regulatory innovation, and lineage-specific evolutionary shifts. TE insertions influence traits such as flowering time, fruit firmness, pigmentation, and the breakdown of self-incompatibility through mechanisms including promoter modulation, methylation changes, and exon disruption. We examine TE-derived small RNAs in epigenetic regulation and summarize evidence for recent TE bursts linked to hybridization, demographic bottlenecks, polyploidization, and local adaptation, collectively contributing to the diversification of *Prunus* genomes. Beyond their biological roles, TE insertion polymorphisms serve as powerful molecular markers for diversity assessment, phylogenetics, and breeding with both retrotransposon- and MITE-based systems showing high discriminatory power across *Prunus* germplasm. Integration of high-resolution TE mapping with functional genomics and epigenetic profiling promises deeper understanding of TE-mediated regulatory networks and their potential exploitation for crop improvement.

## Introduction

Stone fruit trees belong to the genus *Prunus* within the subfamily Prunoideae. The most economically important species include peach [*Prunus persica* L. (Batsch)], almond (*Prunus dulcis* Mill.), apricot (*Prunus armeniaca* L.), sweet cherry (*Prunus avium* L.), sour cherry (*Prunus cerasus* L.), and plums (*Prunus salicina* Lindl., *Prunus domestica* L. among others). According to the latest FAO statistics, global stone fruit production averaged 49.8 million tons in 2023, with peaches contributing nearly half, plums and sloes representing one quarter, and the remainder divided almost equally among apricots, almonds, and cherries [1]. Of the 191 countries listed in the FAO database, 102 cultivate at least one *Prunus* species and only one-third grow five or six species, reflecting the diverse ecological requirements and global economic value of *Prunus*.

Since McClintock’s discovery of Ac/Ds in maize [2], transposable elements (TEs) have been recognized as major drivers of genome evolution, structural diversification and regulatory change [3, 4]. In plants, TE activity has generated lineage-specific genomic signatures and may have contributed to key macroevolutionary transitions, including episodes of rapid diversification during the Cretaceous through insertions carrying transcription factor binding motifs (TFBMs) and conserved non-coding sequences [5]. Similar patterns are evident across the Rosaceae, where shifts in TE composition and activity correlate with phylogenetic branching, mating-system transitions, domestication history, and ecological adaptation. As in other vascular plants, TEs occupy a substantial portion of *Prunus* genomes (Table 1), but their distribution is uneven among species, reflecting distinct evolutionary trajectories and species-specific TE dynamics.

**Table 1:** Percentage of genome occupied by major transposable element classes in *Prunus* species

	*P. yedoensis*	*P. avium*	*P. fruticosa*	*P. campanulata*	*P. mira*	*P. persica*	*P. dulcis*	*P. armeniaca*	*P. mume*	*P. salicina*	*P. cerasifera*
**class I**	**26.4** ^a^	**31.26** ^a^	**30.31**	**29.02**	**36.50** ^a^	**25.18**	**26.5**	**24.97**	**30.97** ^a^	**33.36** ^a^	**25.17**
LTR	24.01	29.03	29.08	23.92	35.61	23.23	24.83	21.16	29.30	31.38	27.93
Ty1/copia	9.5	8.19	7.59	6.54		9.43	9.06	7.48	10.00	9.32	5.31
Ty3/gypsy	14.51	20.84	20.91	8.08		13.8	15.77	13.68	8.60	22.06	17.18
LINE	1.88	1.56	1.23	4.63	0.56	1.5	1.51	1.71	1.62	1.69	0.88
SINE	0.31	0.23	0.19	0.47	0.33	0.29	0.3	0.33	0.05	0.29	0.10
**class II**	**29.74** ^**a**^	**24.83** ^**a**^	**8.19**	**15.47**	**7.24**	**29.24** ^**a**^	**26.5** ^**a**^	**28.82** ^**a**^	**16.01**	**24.48** ^**a**^	**11.86**
CACTA	6.58	4.5				7.41	5.07	4.43		4.25	
MuDR/MULE	7.83	5.68		1.22		7.56	7.16	8.14		6.1	
*FaSt* ^b^	0.13	0.13	0.28	0.18	0.12	0.14	0.19	0.18	0.15	0.12	
PIF-Harbinger	3.24	5.56	1.16	1.47		3.47	3.65	3.73		3.06	
Tc1-Mariner	2.69	2.12	0.07			2.38	2.3	2.82		2.37	
*Helitrons*	3.31	0.34	0.61	0.65		3.04	2.95	3.22		3.12	
**TE total**	**56.14**	**55.81**	**38.69**	**44.49**	**48.38**	**54.41**	**53.31**	**52.28**	**46.19**	**58.05**	**45.93**
Citation	[6]	[6]	[7]	[8]	[9]	[6]⁠	[6]⁠	[6]	[10]	[6]	[11]

Boldface values indicate the total genomic proportion of transposable elements as well as the combined genomic proportions of class I and class II elements; boldface species names indicate those for which standardized TE reannotation was performed on published genome assemblies

^a^Calculated based on published data

^b^Calculated for the present study from BLASTn analyses on *Prunus* genome sequences in Genome Database of Rosaceae (https://www.rosaceae.org/blast)

Beyond contributing to genome size and architecture, TEs are potentially mutagenic; although most insertions are neutral, they may affect gene function and regulation, often generating phenotypic variation relevant to agronomic traits. Variation in fruit quality, development, pigmentation, and stress responses in stone fruits can frequently be traced to TE-mediated insertions or structural changes. This review provides an overview of the structural roles and functional impacts of TEs in stone fruit genomes, with emphasis on cases where transposition has produced phenotypic changes of economic significance and on how TE evolution relates to phylogeny, adaptation, and domestication, and on the development and application of TE-based molecular markers.

### Classification and structure of plant transposable elements

Plant genomes contain diverse transposable elements (TEs) grouped into two major classes following Wicker et al. [12]. Class I elements transpose via a copy-and-paste mechanism using an RNA intermediate. This class includes long terminal repeat (LTR) retrotransposons, LINEs, SINEs, and DIRS-like elements [13]. LTR retrotransposons possess characteristic structural motifs, such as long terminal repeats, primer-binding site (PBS), polypurine tract (PPT), and target site duplications (TSDs). Their replication involves reverse transcription and integration, and the organization of Ty1/copia and Ty3/gypsy elements differs primarily in the position of the *integrase* gene within the *Pol* region and the presence of chromodomains in some gypsy *integrases* [14].

Class II transposable elements transpose without an RNA intermediate. Most class II elements move through a cut-and-paste mechanism mediated by DDE-domain transposases, whereas Helitrons employ a rolling-circle replication strategy [12, 13, 15]. Class II elements typically contain terminal inverted repeats (TIRs) and superfamily-specific TSDs. Major plant superfamilies include CACTA, Mutator-like elements (MULEs), PIF/Harbinger, and Tc1/Mariner. MULEs, including autonomous MuDR elements, are known for capturing and mobilizing gene fragments, contributing to structural and functional genome evolution [16–18].

Non-autonomous derivatives arise from internal deletions of autonomous elements. TRIMs, derived from LTR retrotransposons, retain key LTR-associated signals such as PBS and PPT and often localize near genes, while MITEs represent compact class II derivatives that reach very high copy numbers (0.01% in *Selaginella* to 9.98% in rice; 3.89% in peach) through cross-mobilization by related autonomous elements [19–23]. Given their compact structure, characteristic motifs, and high mobility, these elements are valuable for comparative and marker-based applications in *Prunus*.

These structural and mechanistic properties provide the framework for interpreting TE diversity in *Prunus* and for understanding how specific TE lineages contribute to genome evolution and trait differentiation within the genus.

### Genetic consequences of transposition

The delicate balance between stability and the ability to change has enabled DNA to serve as the engine of evolutionary innovation. Among the forces driving genomic change, the insertion of new genetic material stands out as a major contributor. Fedoroff [24] vividly described transposon activity as stormy “gale-force gusts,” contrasting sharply with the “gentle breeze” of point mutations.

Insertions near regulatory regions can lead to exaptation, introduce cryptic promoters, or modulate expression patterns, and the movement of TEs carrying transcription-factor binding motifs may restructure regulatory networks [5, 25–28]. Likewise, TE activity has contributed to the evolution of complex traits in multiple plant lineages, as demonstrated by TE-mediated redistribution of cis-regulatory elements during the repeated emergence of C4 photosynthesis [29].

Insertions into coding or non-coding regions may disrupt gene function, alter transcript processing, or reshape post-transcriptional regulation [30–33]. For example, MITEs in 3′ UTRs can affect mRNA stability, polyadenylation, or translation and may generate siRNAs influencing other loci [34–36]. Host genomes employ epigenetic and post-transcriptional pathways—including RdDM, siRNAs, and miRNA-mediated regulation—to restrict TE activity, though these mechanisms may also silence nearby genes [37, 38]. Despite such controls, TE-derived mutations continue to accumulate and contribute to functional diversification [21, 39].

These general principles provide an essential framework for interpreting TE-mediated genomic and phenotypic variation in *Prunus*, where lineage-specific TE dynamics and insertions have contributed to distinctive evolutionary patterns and agronomic traits.

### Transposable element composition in *Prunus* genome: methodological constraints and evolutionary tendencies

#### Methodological biases in TE detection

Large-scale genome sequencing of *Prunus* species has revealed that repetitive DNA constitutes a substantial fraction of these genomes, and that earlier estimates consistently undercalled repeat content. This mirrors patterns observed in the human genome, where initial estimates of ~ 50% repeat content were later revised upward to ~ 66–69% [40]. Early assemblies reported modest repeat proportions in *Prunus* (e.g., peach 29.6% [41]; *P. dulcis* 15.88% [42]; *P. avium* 43.8% [43]), but contemporary analyses place most species near or above the 50% threshold (e.g., 54.41% peach; 53.31% almond; 55.81% sweet cherry [6]). Across the *Cerasus* group (*P. yedoensis*, *P. avium*, *P. fruticosa*, *P. campanulata*), total TE content ranges from ~ 38.7% (*P. fruticosa* [7]) to 55.8% (*P. avium* [6]). In *P. armeniaca*–*P. mume* and *P. salicina*–*P. cerasifera* pairs, total TE content generally falls between 52 and 58% [6], with low values in *P. mume* likely reflecting limitations from short-read sequencing [10, 44]. It is further confirmed by the fact that Ty3/gypsy LTR-RTs were consistently the most abundant class I elements across all *Prunus* species, except *P. mume*. Much of this variation likely reflects methodological differences rather than rapid, lineage-specific changes in TE accumulation. *P. cerasifera* currently stands out at ~ 46% despite long-read and up-to-date repeat identification, indicating a need for further analysis [11].

The significant increase in the estimated TE content of the *Prunus* genome is explained by advances in sequencing technology and TE annotation strategies. Short-read assemblies (Illumina/454) often collapsed or underrepresented long, highly similar repeats, particularly in heterochromatin. Long-read technologies (PacBio HiFi, Oxford Nanopore) and improved assembly algorithms now reconstruct repeat-rich regions with high contiguity. Early analyses relied on generic or incomplete TE libraries and homology-only approaches, systematically missing lineage-specific, degenerated, and fragmented elements. In contrast, modern, structure-aware and integrative pipelines (EDTA, RepeatModeler2, REPET/TEdenovo–TEannot, and machine-learning–aided classifiers, e.g., DeepTE) combine structural signatures with homology for greater sensitivity [6, 41, 42, 45]. Species-specific libraries (Dfam, PReDa, PlantRep) further improve classification. Consequently, many TE classes, especially ancient LTR remnants and short, non-autonomous DNA transposons are now recognized at far higher rates. This pattern is exemplified by the genome sequences of *P. fruticosa*, *P. cerasifera*, and *P. mira* [7, 9, 11], in which class I retrotransposons were detected at abundances comparable to those in other related species, whereas class II DNA transposons were markedly underestimated (Table 1).

Advanced, structure-aware pipelines reveal that class II DNA transposons can reach 25–30% in several species, rivaling retrotransposons in genomic share. Reported discrepancies (e.g., *P. salicina* 8.7% DNA TEs in [46] vs. 24.48% in Wang et al. [6]) are best explained by assembly/annotation contrasts rather than intraspecific biology. Long-read assemblies preserve TIRs and TSDs, enabling the detection of both autonomous and non-autonomous elements, including MITEs [22, 47]. Notably, cut-and-paste DNA transposons need not be strictly “self-limiting”: replicative transposition, gap-repair–mediated copy number increases, and episodic bursts can yield substantial long-term accumulation [48–50]. Together, these insights suggest that the contribution of class II elements to *Prunus* genome evolution was historically underestimated and warrants reassessment with standardized methods.

#### Evolutionary shifts in *Prunus* TE landscapes

**Fig. 1:**
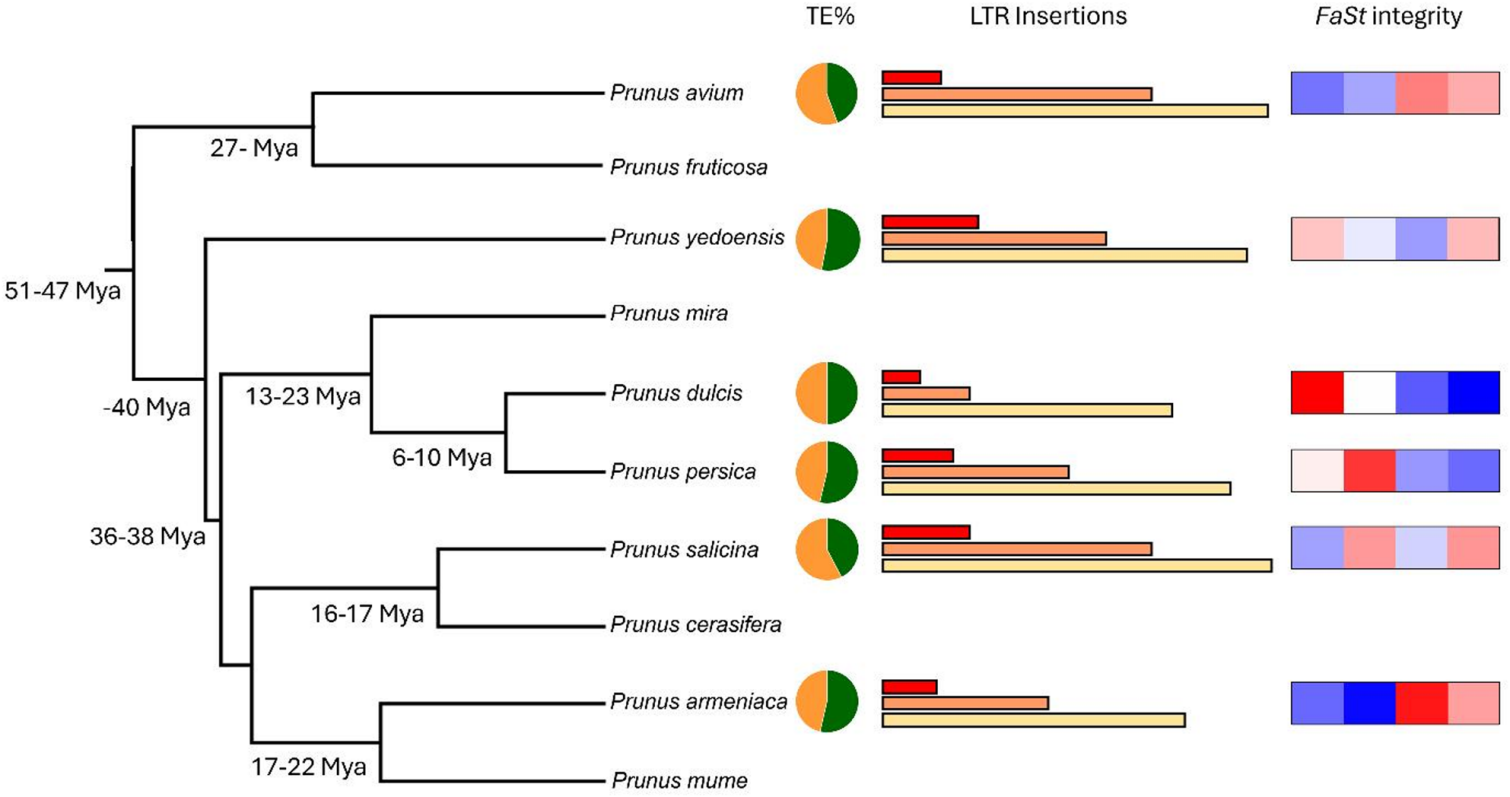
Time-calibrated phylogenetic tree of 12 *Prunus* species generated using TimeTree5 (accessed 01 March 2026). Divergence estimates are shown in million years ago (Mya) at the corresponding nodes, based on Wang et al. [51] and the TimeTree database [52]. The phylogeny provides the evolutionary framework for comparative analyses of transposable element (TE) composition. Pie charts depict the relative proportions of class I (orange) and class II (green) TEs according to Wang et al. [6]. Bar plots illustrate the distribution of long terminal repeat (LTR) retrotransposon insertion ages, with red indicating 0 Mya, orange 0–0.5 Mya, and yellow 0–2.0 Mya, following Wang et al. [6]. The integrity of *Falling Stones* miniature inverted-repeat transposable elements (MITE) is shown as a four-category heatmap (left to right: ≥340 bp; 339–175 bp; 174–82 bp; <82 bp), where color intensity reflects genomic abundance (red = high, blue = low), based on Ivanovska et al. [53]. Original integrity data are provided here for *P. yedoensis*

Standardized re-annotation across six species (*P. yedoensis*, *P. avium*, *P. persica*, *P. dulcis*, *P. armeniaca*, and *P. salicina*) consistently showed that class I elements constitute the largest fraction of TEs in the genomes of *P. salicina* and *P. avium*, whereas the remaining species either exhibit approximately equal proportions of class I and class II elements (e.g., *P. dulcis*) or show a predominance of class II TEs [6]. When TE landscapes were evaluated within a phylogenetic context (Fig. 1), they highlighted lineage-specific shifts as well as patterns consistent with introgressive hybridization, mating system transitions (e.g., the breakdown of self-incompatibility), and domestication-related bottlenecks.

LTR retrotransposons (LTR-RTs) constitute the most abundant class I elements across all surveyed genomes (Table 1) and exhibit a noticeably variable genomic footprint, accounting for approximately 25–33% of total genome size [6]. Many LTR-RT families are shared among species, and substitution-rate analyses by Wang et al. [6] revealed that major expansion pulses occurred predominantly within the past ~ 2 million years (Mya) (Fig. 1). The proportion of the most recent LTR-RT insertions was highest in *P. yedoensis* and *P. salicina*, and lowest in *P. dulcis*. In *P. yedoensis*, the elevated abundance of young LTR-RTs may reflect its hybrid origin (*P. campanulata* × *P. speciosa*) as reported by Song et al. [54]. Hybridization is widely recognized as a trigger of genomic and epigenetic destabilization, including the relaxation of TE-silencing pathways [55], which frequently leads to bursts of LTR-RT activity. A recent LTR-RT burst dated to ~ 0.2 Mya was also documented in *P. salicina* by Huang et al. [56], potentially associated with segmental duplications and frequent gene exchange among trees from diverse geographic regions.

The peach–almond pair (*P. persica* and *P. dulcis*) exhibits a high overall TE content (> 53%) [6]. In both genomes, class II elements occur at proportions equal to or exceeding those of class I, although LTR retrotransposons remain the most abundant superfamily. Phylogenomic evidence places the divergence of peach and almond at ~ 5.88 Mya [57]. Almond has retained a larger fraction of ancestral LTR-RTs, some of which remain polymorphic, whereas peach has lost most ancestral copies, likely due to self-compatibility [58] and reduced effective population size [59, 60]. Nevertheless, peach shows clear signatures of more recent (≤ 5 Mya) retrotranspositional activity, indicating elevated LTR-RT amplification following the species split [57], potentially coupled with its more recent expansion under cultivation [57]. This pattern is consistent with the substantially higher levels of LTR-RT activity observed in peach relative to almond (Fig. 1).

Ty3/gypsy elements are particularly prominent cross *Prunus* genomes. Signals of ongoing transpositional activity, reflected in insertional polymorphism, have been detected at both interspecific and intraspecific levels. In *P. mume*, Ty1-copia (18.4%) and Ty3-gypsy (33.3%) superfamilies represent substantial fractions of the repetitive landscape; although their expression has not been confirmed, purifying selection has been suggested to act on these sequences [61].

**Fig. 2:**
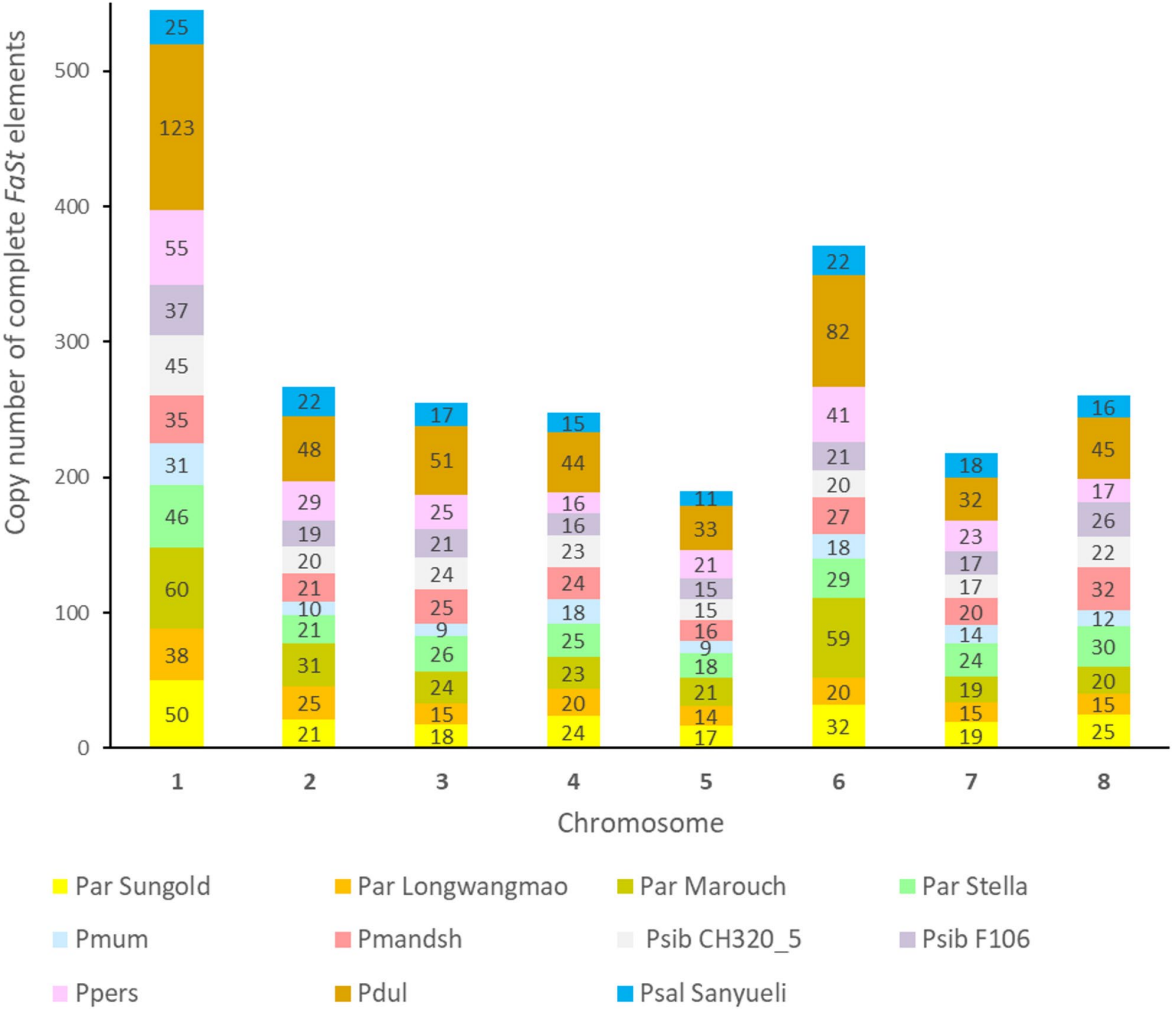
Copy number of *Falling Stone* (*FaSt*) elements across the eight chromosomes (x-axis) in 11 genome assemblies representing seven *Prunus* species. Species abbreviations are as follows: Par, *P. armeniaca*; Pmum, *P. mume*; Pmands, *P. mandshuriaca*; Psib, *P. sibirica*; Ppers, *P. persica*; Pdul, *P. dulcis*; and Psal, *P. salicina*

CACTA and MuDR/MULE elements represent prominent components of the DNA transposon landscape in *Prunus*, consistent with patterns observed in other angiosperms [62], and Helitrons are likewise consistently detected across species. These profiles suggest recent or ongoing mobilization potentially associated with domestication, stress adaptation, or genome restructuring [9, 41, 42]. *Falling Stones* (*FaSt*) MULE elements exhibit pronounced chromosomal bias, with the highest densities on chromosome 1 in peach [30] and comparable enrichment across six additional species (Fig. 2). Chromosomes 1 and 6 generally harbor the largest number of copies, whereas chromosome 5 contains the fewest. Within the *Cerasus* group, *FaSt* elements account for 0.13–0.28% of the genome, reaching their highest proportion in *P. fruticosa*. Relative to total MuDR/MULE content, *FaSt* is particularly abundant in *P. campanulata* (14.8%) but comprises only ~ 2–4% in *P. avium* and *P. yedoensis*, indicating lineage-specific activity or differential retention potentially shaped by genome size variation, epigenetic silencing, or ecological pressures [63].

In peaches, plums, and apricots, absolute *FaSt* abundance remains low (0.12–0.19%) with stable *FaSt*/MULE ratios (~ 1.9–2.7%), a pattern consistent with early expansion followed by long-term suppression typical of non-autonomous elements [64, 65]. Copy-integrity profiles further support this view: fragmented (< 349 bp) sequences dominate in *Cerasus*, whereas *P. yedoensis* retains more complete *FaSt* copies than *P. avium*, and *P. salicina* and *P. armeniaca* harbor comparatively few intact elements (Fig. 1), although at least some apricot *FaSts* have been inferred to remain active [30, 33]. Peach and almond, despite their close evolutionary relationship, show distinct patterns, with *P. dulcis* retaining a greater number of complete *FaSts*—suggesting relatively recent mobilization. Polyploid species carry two- to three-fold more *FaSt* copies, consistent with transposon proliferation during genome duplication [66, 67]. Overall, declining *FaSt*/MULE ratio variability from ancestral to derived lineages, together with progressive structural degradation, mirrors patterns characteristic of other non-autonomous elements such as primate *Alus* [68], which induce mutations through unequal crossing-over and alternative splicing.

Local adaptation provides additional evolutionary context. In Tibetan *Prunus*, SINE expansion is associated with high-altitude environments, with specific insertions colocalizing with loci involved in UV stress responses and the biosynthesis of beneficial metabolites, particularly phenylpropanoids [69]. These findings link TE dynamics to metabolic plasticity and environmental resilience.

A decade ago, repeats in plant genomes were often dismissed as “junk” and routinely filtered out. With the advent of high-quality genome assemblies and improved annotation pipelines, TEs are now recognized as drivers of genome evolution, gene regulation, and adaptative diversification. The apparent expansion of repeat content largely reflects advances in detection and interpretation, underscoring the growing recognition of TEs as dynamic and functionally significant components of plant genomes.

## Phenotypic traits of *Prunus* species associated with transposable elements

Recent years have witnessed the identification of several genes that determine a set of economically important characteristics of stone fruit trees. The measurable evidence of the activity of TEs is the phenotypic traits that could be observed. The movement of TEs in fruit trees could be caused by wounding, pruning, or viral infections [70]. Mutations can arise during gametogenesis, at early stages of zygotic development, or as bud sports in fruit crops. Cross-breeding and the vegetative propagation of woody trees enable the capture and fixation of such novel variants, facilitating the introduction of favorable traits that are critical for crop improvement and commercial profitability. It has been found that about 270 cultivars of peach and nectarines are created as a result of bud sport mutations [71].

### Transposable elements in promoter regions

Accumulating evidence highlights the role of TEs in gene regulatory networks controlling adaptation and flowering time in *P. mume* [72]. MITEs are enriched for TFBMs; and bZIP60 and PIF3-binding sites within MITEs occur frequently near flowering-related genes in the earlier flowering *P. mume* than in *P. persica*. As bZIP60 regulates bud dormancy [73], these patterns suggest that MITEs have rewired these genes in *P. mume* [72].

MITE insertions also underlie key traits in almond, two insertions (including TIR2) upstream of the cytochrome P450 enzyme encoding gene *CYP71AN24* correlate with sweet kernel phenotype (Table 2) by inducing methylation and silencing amygdalin biosynthesis genes [57]. This trait underlies the production of non-toxic, non-bitter edible seeds, a key factor enabling almond domestication. TE insertions near six genes that synergistically have a function in meristem organization, floral development, and fruit cell proliferation, similarly trigger RdDM-mediated silencing [57]. In peach, stony-hard fruit results from a TE insertion upstream of *PpYUC11*, reducing ethylene production [74], while hypermethylation of a TE in the *UDP-ARABINOSE 4-EPIMERASE1* promoter associates with mealiness [75].

**Table 2:** Transposable elements in *Prunus* genomes: classification, insertion sites, and phenotypic effects

Transposon name	Size (bp)	Classification of TE	a. / *n*.a.	Crop species	Insertion site	Consequence	References
−	5,836	class I, LTR, Ty1-copia	a.	*P. persica*	coding region (exon 3) of *PpeMYB25*	nectarine type (hairless) fruit skin	[59]
−	2,569	class II, TIR, CACTA	n.a.	*P. persica*	-1,721 bp of *PpYUC11*	stony hard (non-melting) fruit flesh	[74]
TIR2	−	class II, −, MITE	n.a.	*P. dulcis*	upstream of *CYP71AN24* resulted in high methylation	sweet kernel	[57]
*mMoshan* (*Ds*-like element)	615	class II, *hAT*, MITE	n.a.	*P. cerasus*	coding region of the *S*_1_-*haplotype-specific F-box* gene *SFB*_*1*_	breakdown of self-incompatibility	[76]
*Falling Stone* (*FaSt*)	349	class II, TIR, Mutator, MITE	n.a.	*P. armeniaca*	coding region of the *SFB* gene	breakdown of self-incompatibility	[30]
*Falling Stone* (*FaSt*)	349	class II, TIR, Mutator, MITE	n.a.	*P. armeniaca*	*M*-*locus DsbA-like oxidoreductase*, *Parmdo*	breakdown of self-incompatibility	[33]
−	1,848	−*	n.a.	*P. avium*	-280 bp of *M locus glutathione S-transferase* (*mgst*)	breakdown of self-incompatibility	[77]
LTR1	5,282	class I, LTR, Copia-like	n.a.	*P. persica*	intron of the *PpCCD4* gene	yellow flesh colour	[78]
LTR2	6,204	class I, LTR, Ty1-Copia	a.	*P. ferganensis*	coding region, at + 2433 bp (3rd exon) of *PfCCD4* gene	yellow flesh colour	[79]
*mMoshan*	588	class II, *hAT*, MITE	n.a.	*P. persica ‘Hongyetao’*	526 bp upstream of the ATG translation start codon of *PpCHI*	over 100-fold higher promoter activity of *PpCHI*	[80]
*mMoshan*	590	class II, *hAT*, MITE	n.a.	*P. persica* self-pollinated progeny of ‘Malo Konare’	+ 1168 bp (3rd exon) of *PpLMI1* gene (*Prupe 7.G121100*)	absence or globose phenotype of extrafloral nectaries	[81]
–	6,300	class I Copia-like	–	*P. persica*	first intron of *PpeDAM5*	low-chill phenotype	[82]⁠
	2,600	class II CACTA	n.a.	*P. persica*	first intron of *PpeDAM6*	low-chill phenotype	[82]⁠
–	155	–	n.a.	*P. persica*	downstream from + 979 to + 1133 in *SFB*^*1*^	breakdown of self-incompatibility	[83]
–	2,615	class II, TIR, MULE	–	*P. cerasus*	5’ flanking region (-797) of the *S*_*6*_*-RNase*	breakdown of self-incompatibility	[84]⁠
–	6,800	–	–	*P. mume*	coding region, at + 534 bp of *SFB*^*f*^	breakdown of self-incompatibility	[85]⁠
–	–	class I, SINE	n.a.	*Wild Tibetan P. mira*, *P. mume*, *P. armeniaca*	phenylpropanoid pathway genes	high altitude adaptation	[61]
–	–	–	–	*P. persica*	*CYP82A3* and *UDP-arabinose 4*	mealiness	[75]
–	–	class I Copia-like and a Helitron		*P. mume*	5’ flanking region (-2 kb) of the *Pm031359 (UGT79B6)* gene	white petal color	[86]
–	–	class I, 2 L1 and a Copia		*P. mume*	*Pm013782 (DFRA)*	white petal color	[86]

*According to the features, most probably a MULE (9-bp TSD, 300-bp TIR) [20]

– no data

Flower color chimerism in *P. mume* links to TE insertions with differently methylated regions (DMRs) upstream of genes involved in pigment biosynthesis [86]. TE-adjacent lncRNAs identified in a newly assembled Rosaceae plant TE database (RPTEdb) further implicate RdDM in floral variation [87]. The *mMoshan* MITE upstream of peach *chalcone isomerase PpCHI* (Table 2) dramatically enhances promoter activity (> 100-fold) by introducing MYB-binding sites [80].

Mining genomic sequence databases continues to reveal TE-driven cis-regulatory changes. For example, a *FaSt* element in the almond *C-repeat binding factor PdCBF1* promoter harbors WRKY motifs [88], potentially explaining differential cold response compared to *PdCBF2* [89]. A *FaSt* insertion upstream of an *S*_C_-*RNase* allele in apricot (unpublished data) shows no transcriptional effect [90, 91], leaving its functional role unresolved.

### Transposable elements in 5’ untranslated regions

While MITE and other TE families have been annotated across *Prunus* genomes and are often found within genic regions including UTRs in silico, there are only a limited number of reports that explicitly characterize a TE insertion within the 5’ UTR of a *Prunus* gene. Although *Moshan* and other TEs were reported in 3′ UTRs [80], comparable analyses of 5’ UTR insertions were largely precluded by incomplete annotation of transcription start sites at the time. Consequently, potential 5′ UTR insertions may have been subsumed under promoter or first exon categories rather than explicitly annotated. However, Ivanovska et al. [53] identified three *FaSt* elements within 5’ UTRs of some plum and almond genes that are associated with key physiological processes, such as fruit softening, no direct evidence currently supports a functional impact of the *FaSt* insertions on gene activity.

### Transposable elements in coding exons

A MITE of the *hAT* superfamily (mMoshan) was identified in the third exon of the peach gene coding for a Late Meristem Identity (*LMI1*) TF [81]. It was associated with the absence or globose phenotype of extrafloral nectaries. The insertion of the transposon was positively correlated with enhanced expression of the gene putatively due to the *cis*-regulatory elements such as MYB and WRKY binding sites in the first third of the sequence [80].

The insertion of an LTR retroelement in exon 3 of the *PpeMYB25* gene of peach induces the recessive glabrous phenotype, the nectarine phenotype, a highly demanded trait enhancing consumer acceptance [92]. In peach, yellow flesh is another important commercial trait, and an LTR-type TE (LTR2) has been identified within an exon of *PpCCD4*, which encodes a carotenoid cleavage dioxygenase responsible for carotenoid degradation. The insertion is associated with loss of *PpCCD4* function, likely reducing carotenoid cleavage and contributing to the yellow flesh phenotype [79].

### Transposable elements in introns

Yellow flesh color was shown to originated independently through multiple mutational mechanisms affecting the same *PpCCD4* gene [78]. Besides LTR2 in the exon, a similar LTR retrotransposon insertion (LTR1) within the single intron of the *PpCCD4* gene was shown to be associated with altered gene expression, likely through transcriptional interference or disrupted splicing, thereby contributing to stable suppression of carotenoid cleavage (Table 2).

Naturally, all *Prunus* fruit trees go into dormancy during the winter period, but those considered as ever-growing, have no perception of the short days, lower temperature, and are not forming terminal vegetative buds [93]. A fragment of an autonomous retrotransposon has been identified within the exon of the polyprotein-encoding gene *EVG10* and is implicated in this trait (Table 2). Copia-like and CACTA-insertions were identified in the first intron of *Dormancy-Associated MADS-box* genes *PpeDAM5* and *PpeDAM6*, respectively, only in low-chill peach cultivars, resulting in lower levels of gene expression than in high-chill cultivars [82]. The higher expression of *DAM6* gene was also shown to result in late flowering in other species like apricot [94].

In *P. persica*, 31, 10, and 6 miRNAs originated from the *Mutator*, *hAT*, and *PIF*/*Harbinger* transposon superfamilies, respectively, with approximately one third of these elements located within intronic regions [95]. These findings support a transposition–transcription mechanism through which MITEs give rise to novel miRNAs: insertion into introns of transcriptionally active genes enables MITEs to be transcribed and subsequently recognized by the miRNA biogenesis machinery. Notably, genes targeted by MITE-derived miRNAs were predominantly involved in responses to environmental cues, including temperature, indicating that MITE-miRNAs may contribute to plant adaptive processes. Altogether, the molecular domestication of MITEs represents a genomic strategy that facilitates rapid and ongoing diversification of miRNA repertoires in angiosperms.

### Transposable elements in 3’ untranslated regions

Among the 29 *mMoshan* and 10 *tMoshan* elements identified within *P. persica* gene sequences, three and one insertions, respectively, were in 3′ UTRs [80]. Similarly, in *P. domestica*, the 3′ UTRs of the *ethylene-responsive transcription factor* and *glucosyltransferase* genes were found to harbor *FaSt* element insertions [53].

## Transposable elements causing the breakdown of self-incompatibility

The genetic diversity in angiosperms is preserved by outcrossing ensured by the gametophytic self-incompatibility (SI) [96, 97]. To avoid inbreeding depression, fruit trees have developed a ribonuclease enzyme based molecular system to prevent self-pollination [98]. The multiallelic *S* locus is gametophytically expressed and is responsible for SI in *Prunus sp*. by having a pistil-expressed *S-ribonuclease* (*S-RNase*) gene and a pollen-expressed *S-haplotype-specific F-box* (*SFB*) gene (Table 1) [99, 100]. Sexual compatibility is regulated by the haploid genome of the pollen and the diploid genome of the pistil. The recognition between self- non-self-pollen depends on the identification of the *S*-allele product in the pollen by the two *S*-allele products in the pistil in diploid species. In case of self-compatible plants, the pollen carrying the same *S*-allele as in the pistil can pollinate the flower due to loss-of-function of either or both of the molecular partners [101]. Many of those non-functional mutations have been described to be induced by TEs (Table 2).

The sour cherry *S*_6m_-RNase of the ‘Érdi bőtermő’ cultivar has an approximately 2,615 bp insertion in the 5’ flanking region at -797 of the *S*_6_*-RNase*, which results in the loss of transcription of the gene [84]. A *Ds*-like element, 615 bp long, has been found in the coding sequence of *SFB*_1_ gene termed *S*_1_’ [76]; this insertion introduces a premature stop resulting in a putative truncated SFB_1_. This TE was later identified as a representative of *Moshan* elements, characterized by 11–13 bp TIRs and 8-bp TSDs [80]. The mutated sour cherry *S*-haplotypes were found as non-mutated, wild-type *S*-haplotypes in sweet cherry, the diploid progenitor species of *P. cerasus*. Sour cherry as a recent polyploid is hypothesized to experience bigger transpositional activity and chromosomal rearrangement, described as genome instability [66, 102]. Likewise, the polyploidization presents paralogous copies of each *S*-haplotype, which speeds up the rate of evolution [66] without selection pressure, since any mutation remains latent until the accumulation of at least two non-functional *S*-haplotypes that can break through the incompatibility barrier in the tetraploid sour cherry [103].

In four *P. cerasus S*_36_ variants, a 306 bp non-autonomous *Helitron* element was detected in the 3’ UTR (38 bp downstream of the *SFB* stop codon) [104]. The polyadenylation signal required for transcription termination is located within the *Helitron* sequence, downstream of a motif that may function as a transcriptional pause or termination signal. Consequently, a fraction of the resulting transcripts was shown experimentally to lack proper polyadenylation, which may reduce mRNA stability and interfere with efficient protein synthesis.

In *P. mume*, the *S*_f_-haplotype harbors a non-functional *SFB* gene caused by a 6.8-kb insertion within the coding region at position + 534, resulting in truncated proteins that lack the hypervariable regions required for allele-specific recognition [85]. The 396-bp sequences at both ends of the insertion are identical, consistent with the LTRs of a retrotransposon. A 5 bp insertion in the *SFB*_2_ gene in peach resembles the footprint of a transposon, that has introduced a premature stop codon [83].

**Fig. 3:**
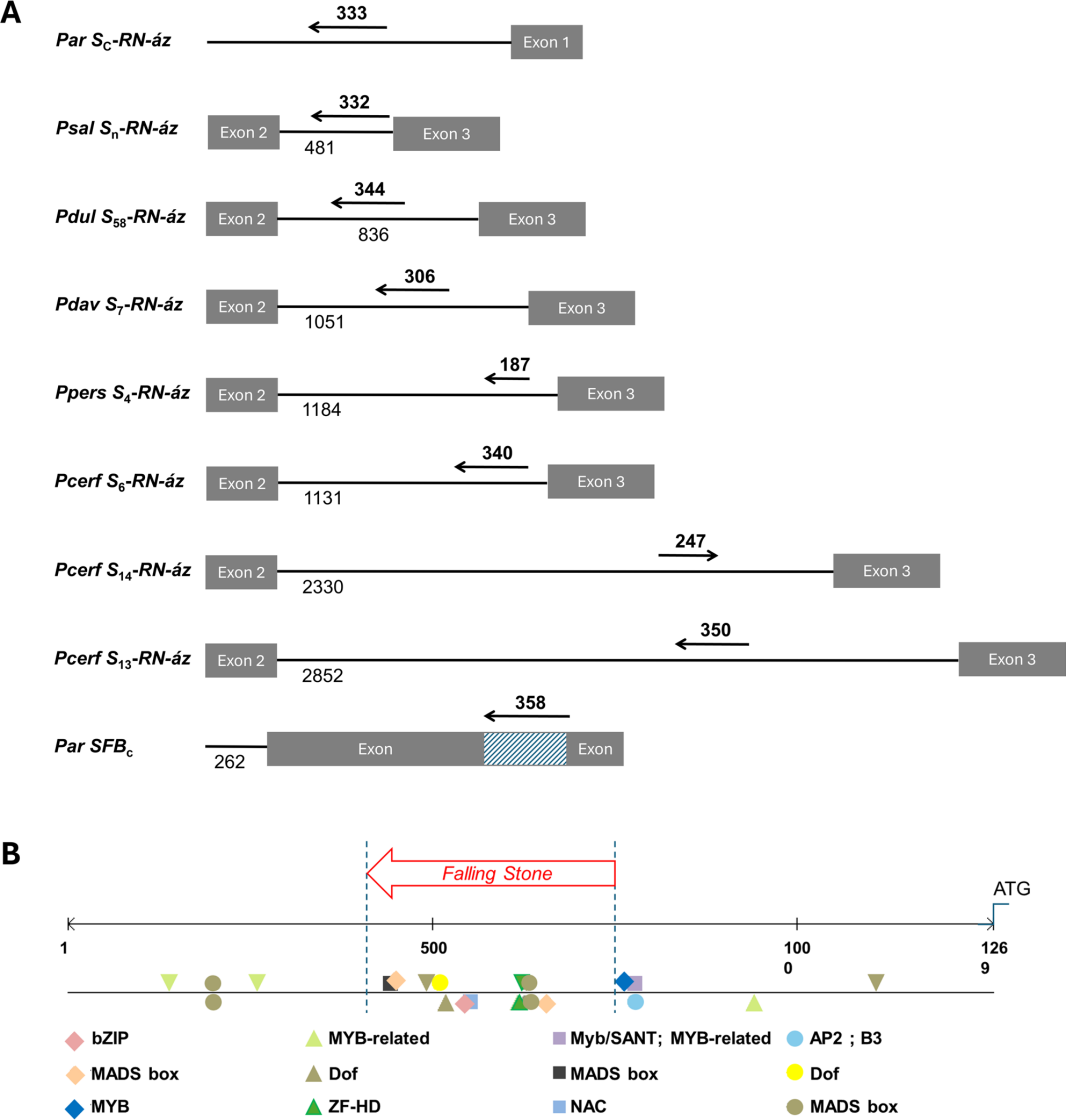
*Falling Stones* (*FaSt*) miniature inverted-repeat transposable elements in the self-incompatibility locus of *Prunus* species. **A** Positions and orientations of *FaSt* elements within promoters, introns (lines), and exons (grey boxes). Arrows and diagonally hatched boxes indicate element location and orientation; element sizes (bp) are shown above the arrows. **B** A *FaSt* element (red arrow) in the *Prunus armeniaca S*_C_-*RNase* promoter contains multiple predicted transcription factor binding motifs

The non-autonomous *Mutator* MITE element, *FaSt*, identified in apricot was shown to be responsible for the self-compatible phenotype [30]. This 349 bp MITE element is inserted into exon 3 of the *SFB*_8_ allele, that results in the formation of a truncated protein lacking the HVa and HVb regions [105, 106]. Moreover, the same MITE transposon *FaSt*, has been located at the *M*-locus of chromosome 3, known as a pollen modifier gene in apricot [33, 106]. Though the *Par-Mm*-allele is outside the *S*-locus, it is still responsible for GSI breakdown in the apricot cultivars ‘Canino’ and ‘Katy’ (Table 2) [107]. It disrupts the function of the gene encoding for disulfide bond A-like oxidoreductase protein *Par*MDO, by insertion in the ORF, therefore 4 exons are missing [33]. *FaSt* elements were also detected in the promoter and intron regions of the *S*-locus genes of many *Prunus* species (Fig. 3).

Recent findings have confirmed that ‘Cristobalina’, a SC sweet cherry cultivar does not carry non-functional mutation at the *S*-locus [77, 108]. A transposon-like insertion was shown at the promoter region of the *M* locus-encoded *glutathione S-transferase* (*MGST*) gene, located on the edge of the chromosome 3, a similar region as *mdo* in apricot [77].

## Transposon-based molecular marker analysis in *Prunus* fruit tree species

**Fig. 4:**
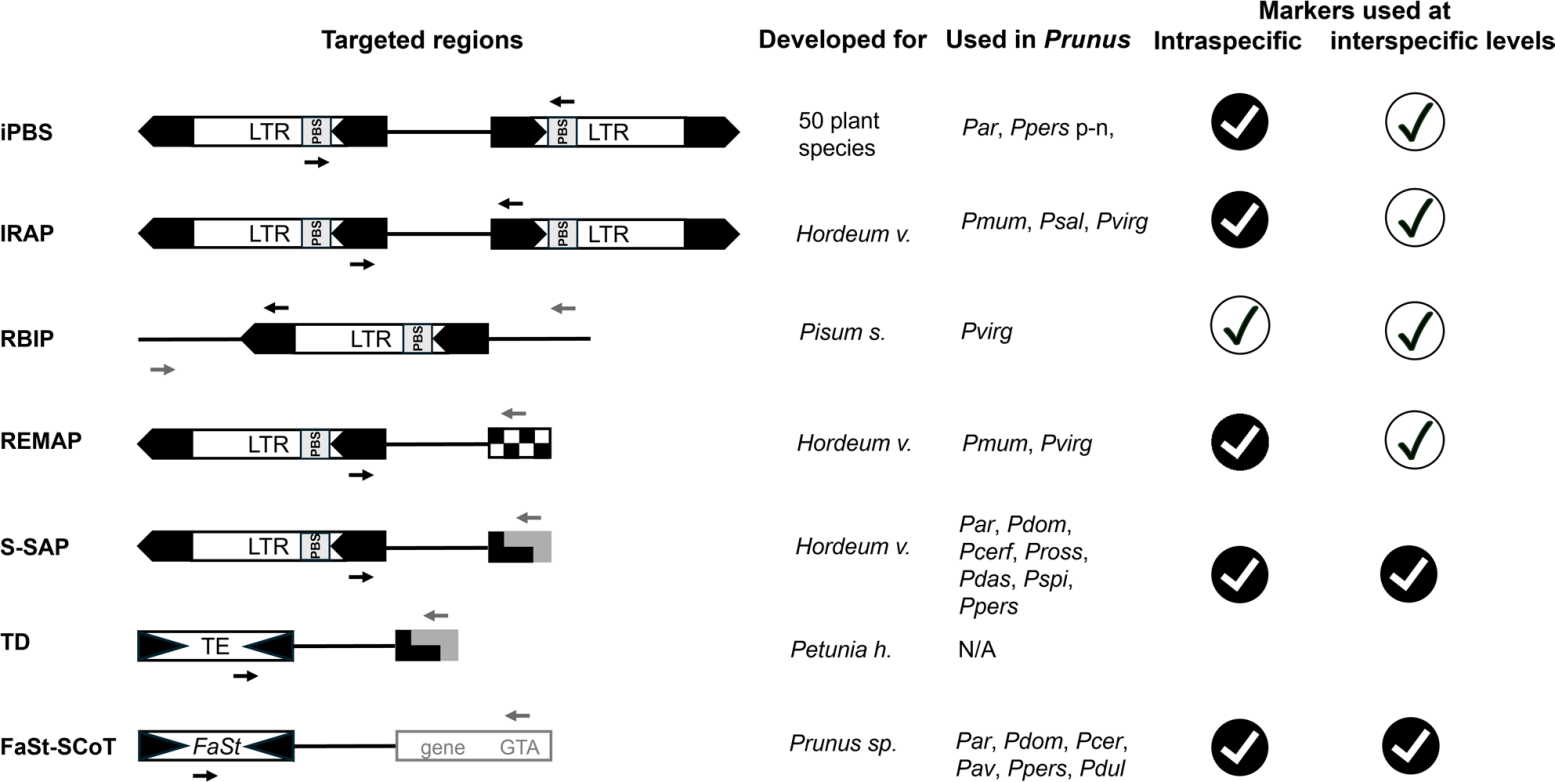
Transposable element (TE)-based molecular markers applied in the analysis of *Prunus* species. Marker types: iPBS (inter-primer binding site), IRAP (inter-retrotransposon amplified polymorphism), RBIP (retrotransposon-based insertion polymorphism), REMAP (retrotransposon-microsatellite amplified polymorphism), S-SAP (sequence-specific amplification polymorphism), TD (transposon display), and FaSt-SCoT (*Falling Stone*–start codon targeted). Symbols: black pentagons represent long terminal repeats (LTRs) of retrotransposons; black triangles indicate terminal inverted repeats of DNA transposons; grey squares denote primer binding sites (PBS); checkered boxes correspond to simple sequence repeats (SSRs); black lines show intervening DNA regions; black arrows indicate PCR primers designed from TE sequences; grey arrows indicate primers designed from flanking non-TE regions. Species abbreviations: *Par* (*P. armeniaca*), *Pcer* (*P. cerasus*), *Pcerf* (*P. cerasifera*), *Pdas* (*P. dasycarpa*), *Pdom* (*P. domestica*), *Pdul* (*P. dulcis*), *Pmum* (*P. mume*), *Ppers* (*P. persica*), *Pross* (*P. rossica*), *Psal* (*P. salicina*), *Pspi* (*P. spinosa*), *Pvirg* (*P. virginiana*), and N/A (not applied). Verification symbols: white tick on black background indicates experimentally validated use; black tick indicates hypothesized application

 The insertional polymorphisms of TEs have been increasingly exploited for molecular marker development. In economically important *Prunus* fruit tree species, retrotransposon and transposon-based markers offer a powerful tool for assessing genetic diversity, phylogenetic relationships, and evolutionary dynamics. Retrotransposons, particularly those with LTRs, are the most used source for TE-based dominant markers due to their high copy number and widespread distribution. Figure 4 illustrates TE-based marker systems such as IRAP (inter-retrotransposon amplified polymorphism), inter-primer binding site (iPBS), REMAP (retrotransposon-microsatellite amplified polymorphism), and RBIP (retrotransposon-based insertion polymorphism), which have been widely applied in plants to detect insertional polymorphisms and infer genetic relationships [109]. IRAP and iBPS are single primer approaches with primers designed from the conserved regions of LTR or PBS [110]. RBIP is the only codominant marker resulting in the amplification of differently sized fragments based on the absence or presence of a retrotransposon. The rest of the markers uses a combination of retrotransposon-specific primers with other markers like microsatellites or simple sequence repeats, SSRs (REMAP), amplified fragment length polymorphism, AFLP (sequence-specific amplification polymorphism, S-SAP and transposon-display, TD) or start-codon targeted markers, SCoT (FaSt-SCoT).

For the REMAP analysis of Japanese apricot cultivars, five LTR primers were combined with four SSR primers, yielding an average of 24.4 bands per primer combination [111]. Six LTR primers were used for IRAP amplification, which provided an average number of 16.5 polymorphic bands per primer. Fruit-bearing and ornamental cultivars clustered into different groups which confirmed the differences in the origin and evolutionary history of the corresponding germplasm. IRAP markers were also used for the genetic analysis of *P. salicina* ‘Shazikongxinli’, which proved to be more polymorphic than inter simple sequence repeat (ISSR) markers [112]. The authors concluded that retrotransposons are more suitable for intraspecific variability detection than SSR regions. Liang et al. [113] developed 283 L-based markers for chokecherry (*P. virginiana*) and demonstrated their utility in genetic mapping as well as their transferability to related species.

The iPBS marker uses primers that anneal to a tRNA sequence present in LTR retrotransposons [114]. Naeem et al. [115] applied 9 polymorphic primer pairs to estimate the genetic diversity and population structure of 48 peach and nectarine genotypes from various geographical regions of Pakistan, and data illustrated the rather limited germplasm exchange among these regions.

The AFLP method was adapted to assess TE insertion polymorphisms by employing LTR-based primers for PCR amplification (referred to as sequence-specific amplified polymorphism, S-SAP [116] or primers for DNA transposons (known as TD [117], . Six and 22 primers were used for the S-SAP and iBPS analyses of 28 Velkopavlovická (syn. Magyarkajszi) apricot clones [118]. The study did not confirmed that TE-based markers were more suitable for distinguishing among clones that were in general very similar to each other regardless of their origin. The S-SAP analysis of *P. domestica* and related species indicated *P. domestica* had an isolated position. This might be explained by a more complex origin of the species or the large-scale genomic rearrangements due to the activation of TEs during polyploidization [119].

Most TE-based marker assays conducted in *Prunus* species rely on retrotransposon-derived markers. However, DNA transposons, especially non-autonomous elements like MITEs have also gained attention for their proximity to genes and potential regulatory impact. A recent study by Ivanovska et al. [53] introduced a novel marker system combining start-codon targeted (SCoT) primers with a transposon-specific primer targeting the *FaSt* MITE. This combined SCoT–FaSt approach was tested across 12 cultivars from six *Prunus* species and 28 cultivars of European plum (*P. domestica*). Compared to SCoT-only assays, the combined system produced fewer total bands but a higher percentage of polymorphic bands, with comparable values for polymorphism information content (PIC), resolving power, and gene diversity. The broader size range of amplified fragments improved gel resolution and facilitated the detection of differentially amplified loci. In addition, sequencing of selected SCoT–FaSt amplicons revealed that *FaSt* elements were frequently located in UTRs and introns of genes with key physiological functions. This suggests that *FaSt* insertions may influence gene expression and contribute to phenotypic variation.

TE-based markers (Fig. 4) are most frequently applied across both intra- and inter-specific taxonomic levels, though some of the markers (e.g. iPBS) may be used in very distantly related plants, from pteridophytes to angiosperms. In *Prunus*, most of the TE-based markers were used to understand the genetic structure of populations or differentiate among cultivars. Though RBIP markers were designed and used in chokecherry and other species, intra- and inter-specific polymorphism was not detected on gels [113]. It is expected that many of those markers are also informative at interspecific levels, but relevant studies are limited so far.

Phylogenetic analyses based on SCoT–FaSt markers successfully resolved interspecific and intraspecific relationships [53]. For example, *P. persica* and *P. dulcis* formed a well-supported cluster, reflecting their close evolutionary relationship. In contrast, cultivars of *P. domestica* and *P. armeniaca* grouped separately, and members of the *Cerasus* subgenus formed distinct clusters. Principal component analysis further confirmed the genetic structure among plum cultivars, distinguishing modern German cultivars from Hungarian landraces and older international cultivars.

In summary, transposon-based molecular markers, particularly those targeting non-autonomous elements, offer a versatile and informative approach for genetic analysis in *Prunus*. Their ability to detect polymorphisms, combined with high-throughput sequencing and bioinformatics tools, positions them as valuable assets in breeding, conservation, and evolutionary studies.

## Conclusions

TEs in *Prunus* species have shifted from being considered genomic “junk” to recognized engines of evolutionary innovation and phenotypic diversity. They occupy over half of most *Prunus* genomes and actively shape traits of agronomic importance, from fruit quality to sexual compatibility. Their dynamic activity not only drives structural variation but also rewires regulatory networks, influencing adaptation and domestication. TE-derived polymorphisms offer practical tools for breeding and genetic studies, reinforcing their value in application. While considerable progress has been achieved, current understanding remains limited by species-specific annotation biases, incomplete functional validation of TE insertions, and methodological differences across published genome assemblies. Looking ahead, coupling high-resolution TE maps with functional and epigenomic analyses will illuminate how these elements orchestrate gene regulation and stress responses. Such insights will enable breeders to harness TE-driven variability for resilience and crop improvement. Whirlwinds may scatter stones, but “the stronger winds of transposon activity” [24] have the power to reshape *Prunus* genomes.

## Data Availability

No datasets were generated or analysed during the current study.

## Abbreviations

CBF: C-repeat binding factor
CCD4: Carotenoid cleavage dioxygenase
CHI: Chalcone isomerase
DAM: Dormancy-associated MADS-box gene
DIRS: Dictyostelium intermediate repeat sequence
*FaSt*: *Falling Stones*
iPBS: Inter-primer binding site
IRAP: Inter-retrotransposon amplified polymorphism
ISSR: Inter simple sequence repeat
LINE: Long interspersed elements
LTR: Long terminal repeat
LTR-RT: LTR retrotransposons
MDO: Disulfide bond A-like oxidoreductase
MGST: Glutathione S-transferase
MITE: Miniature inverted repeat transposable elements
MULE: Mutator-like elements
Mya: Million years ago
PBS: Primer binding site
PPT: Polypurine tract
RBIP: Retrotransposon-based insertion polymorphism
RdDM: RNA-directed DNA methylation
REMAP: Retrotransposon-microsatellite amplified polymorphism
SCoT: Start-codon targeted markers
SFB: S-haplotype-specific F-box
SI: Self-incompatibility
SINE: Short interspersed elements
S-RNase: S-ribonuclease
S-SAP: Sequence-specific amplification polymorphism
SSR: Simple sequence repeats
TD: Transposon-display
TE: Transposable element
TIR: Terminal inverted repeats
TPase: Transposase
TFBM: Transcription factor binding motifs
TRIM: Terminal-repeat retrotransposons in miniature
TSD: Target site duplications
UTR: Untranslated regions
